# Impact of Clouds and Blowing Snow on Surface and Atmospheric Boundary Layer Properties Over Dome C, Antarctica

**DOI:** 10.1029/2022JD036801

**Published:** 2022-11-10

**Authors:** Manisha Ganeshan, Yuekui Yang, Stephen. P. Palm

**Affiliations:** ^1^ Morgan State University Baltimore MD USA; ^2^ NASA Godddard Space Flight Center Greenbelt MD USA; ^3^ Science Systems and Applications, Inc Lanham MD USA

**Keywords:** CALIPSO, blowing snow, Antarctic boundary layer, Antarctic clouds, surface downwelling longwave radiation, surface‐based inversion

## Abstract

Clouds and blowing snow (BLSN) occur frequently over Antarctica, where it is critical to understand their feedbacks to surface and atmospheric boundary layer processes. Dome C, an elevated East Antarctic station, dominated by lengthy periods of surface longwave (LW) radiative cooling, is selected to reveal cloud and BLSN impacts within a largely stable environment. The sky condition is classified as clear, cloudy, or BLSN, using 3 years of Cloud‐Aerosol Lidar and Infrared Pathfinder Satellite Observations satellite data. Co‐located and contemporaneous in situ observations are used to investigate the relationship of sky condition with surface and atmospheric boundary layer thermal structure, focusing on seasonal variability. Results show that increased downwelling LW radiation from clouds abate surface radiative cooling losses, contributing to warming during all seasons. An increase of 3°C in the mean surface air temperature is observed during spring, whereas, a more dramatic rise (around 10°C), due to accompanying large‐scale subsidence, is observed during fall and winter in association with clouds. For all seasons, the wind speed and wind speed shear are strongest during BLSN events, and the surface‐based inversion is weakened by cooling which peaks in a shallow above‐surface turbulent layer. The stronger background stability during fall and winter seasons, restricts turbulence and BLSN depths generally to the lowest tens of meters. The Earth's cryosphere is among the most rapidly evolving yet least well‐observed regions, and knowledge of clouds and BLSN interactions with the typical stable atmospheric boundary layer can help further understand energy and moisture exchanges.

## Key Points

- Cloud‐Aerosol Lidar and Infrared Pathfinder Satellite Observations and co‐located in situ observations allow study of cloud and blowing snow (BLSN) impacts on Antarctic atmosphere
- At Dome C, clouds abate surface longwave radiative cooling losses, causing warming in all seasons
- BLSN, accompanied by intense winds, weakens the temperature inversion, and is vertically restricted by strong background stability

## 1 Introduction

The atmospheric boundary layer over Antarctica, especially East Antarctic plateau, often features a stable surface‐based inversion (SBI) owing to the prolonged net radiation deficit occurring over the icesheet for most of the year. Accurate knowledge of the boundary layer thermodynamic structure is deemed vital for polar regions (NASEM, 2018; Teixeira et al., 2021), and the difficulty in parameterizing turbulence in stable conditions (Couvreux et al., 2020; Sandu et al., 2013) can affect the representation of Antarctic climate in global models and reanalyses (Ganeshan & Yang, 2019; Sandu et al., 2013).

Two important factors that interact with, and therefore influence, the Antarctic surface temperature and boundary layer thermodynamic structure are clouds and blowing snow (BLSN) (e.g., Hudson & Brandt, 2005; Zhang et al., 2011). Antarctic clouds play a role in the surface radiation budget in addition to contributing to its mass balance through precipitation processes. A number of observational studies have attempted to quantify the net positive (warming) impact of Antarctic clouds, also known as Cloud Radiative Effect or CRE (Aoki & Yamanouchi, 1992; Pavolonis & Key, 2003; Scott et al., 2017; Town et al., 2005). Through modeling experiments, there is further evidence that Antarctic CRE can modify the atmospheric circulation, which in turn, affects local and global climate (Lachlan‐Cope, 2010; Lubin et al., 1998). Lawson and Gettelman (2014) have found much higher than anticipated occurrence of low‐level mixed phase clouds over the South Pole, which when accounted for in a global modeling system, significantly increases the CRE, and likely has an impact on the atmospheric boundary layer.

Past observations have primarily studied Antarctic cloud impacts at the surface (and top of atmosphere), with a few that have explored direct connections with the atmospheric boundary layer. For example, during a case study, Hudson and Brandt (2005) found that the SBI over the South Pole was nearly destroyed due to increased downwelling longwave (LW) radiation associated with the passage of a cloud. Similarly, Pietroni et al. (2014) found that SBI strength over Dome C can be reduced in the presence of cloud cover. On the other hand, through an investigation of dropsondes from the Concordiasi campaign during austral spring season, Ganeshan and Yang (2018) found that regions with maximum cloud cover frequency over West Antarctica also had a maximum occurrence frequency of well‐mixed boundary layers, which they speculated was due to frequent occurrence of synoptic disturbances that could contribute to cloudiness and boundary layer turbulence alike.

In addition to the presence of clouds, the sky condition, especially over East Antarctica, is uniquely characterized by BLSN which occurs 50% of the time or greater during the cold season (April–October; Palm, Kayetha, & Yang, 2018; Yang et al., 2021). This phenomenon plays a significant role in Antarctica's surface mass balance by sublimating and redistributing snow across the continent, and like clouds, may also impact the surface radiation and atmospheric boundary layer. The radiative impact of Antarctic BLSN has been previously quantified using satellite observations (Yang et al., 2014).

One possible pathway of interaction between BLSN and atmospheric boundary layer is elaborated below. Using dropsonde observations from the Concordiasi campaign, Palm, Yang et al. (2018) found that for the austral spring season, BLSN events over Antarctica are typically accompanied by an isothermal or well‐mixed boundary layer because of turbulent mixing due to strong wind shear related to both vertical gradients in wind direction and speed. Finding similar evidence of well‐mixed spring‐time Antarctic boundary layer during the Concordiasi campaign, Ganeshan and Yang (2018) concluded that mixing is chiefly mechanically produced due to increased low‐level wind speed shear.

However, unanswered questions regarding boundary layer mixing in the presence of BLSN still exist. We can use observations at Dome C station to address some of these questions. Dome C (75.10°S, 123.35°E) is located on the Antarctica Plateau at an elevation of 3,233 m above sea level. The low‐level wind speeds over Dome C are generally calm (<4 m s^−1^) to moderate (4–6 m s^−1^), with rare occurrence of strong winds (∼10% frequency; Petenko et al., 2019). Moreover, katabatic winds, which lead to boundary layer mixing (Ganeshan & Yang, 2018) are not usually observed over Dome C (Genthon et al., 2021). In fact, due to the strong radiative cooling at altitude and relatively undisturbed conditions, the atmospheric boundary layer over Dome C is most likely always stable, except during periods of maximum insolation in summer, when a well‐mixed or convective boundary layer regularly occurs during the peak hours of local afternoon (Argentini et al., 2005, 2014; Casasanta et al., 2014; Gallée et al., 2015; King et al., 2006). Therefore, during the cold season over Dome C, very few, if any, mixed boundary layers are expected. On the other hand, BLSN occurs most commonly during the winter months, and at least as frequently as 10%–20% of the time over Dome C (Palm, Kayetha, & Yang, 2018). The boundary layer structure and properties during winter time BLSN events, thus warrant investigation.

Recently, using advanced high‐resolution sodar, Petenko et al. (2019) discovered the existence of significant turbulence over Dome C during winter, extending several tens of meters, in spite and (a) in the presence of very low surface temperatures and large static stability (strong temperature inversions extending up to 100–600 m and with inversion strength reaching 20–40°C), (b) the absence of orographic features (and strong katabatic winds), and (c) the absence of the diurnal cycle of solar heating (insolation). They termed these turbulent layers as surface‐based turbulent layers (SBTL) that occur embedded within the winter SBI at Dome C (unlike and more complex than the summer time convective boundary layer). SBTLs were found to occur during synoptically disturbed conditions as well as during calm and moderate winds without large‐scale background forcing albeit with differences in their vertical structures and organization. When it occurs in the absence of synoptic disturbances, the SBTL height depends linearly on the low‐level wind speed above a certain threshold, and is negatively correlated with near‐surface stability (i.e., temperature difference between 10 m level and surface). In this study, evidence for the occurrence of both, mixed layers and SBTLs will be investigated during BLSN conditions at Dome C.

Overall, our knowledge regarding the relationship between clouds, BLSN, and boundary layer thermal structure over Antarctica, especially its seasonality, remains incomplete. In this study, we will conduct investigations using surface‐based measurements at Dome C, in conjunction with data from the Cloud‐Aerosol Lidar and Infrared Pathfinder Satellite Observations (CALIPSO) satellite, which will be used for scene classification. Dome C is selected because it offers a comprehensive suite of continuous, long‐term ground‐based atmospheric measurements and is also covered by critical satellite observations which can be combined to study the surface and atmospheric boundary layer properties as a function of clear, cloudy, and BLSN sky condition. Dome C, due to its remote, elevated location away from the coast, has milder volatility in synoptic patterns, and is therefore ideal to investigate seasonal modes of variability in the relationship of clouds and BLSN with the surface and atmospheric boundary layer. One caveat, however, is that it lies in an atypically flat region of the interior ice sheet where there is little to no downslope buoyancy forcing (and katabatic winds).

The following section outlines the various datasets that will be used in this analysis, and the method of classifying the scene as clear, cloudy, or BLSN.

## 2 Materials and Methods

One of the challenges in studying interactions of Antarctic clouds and BLSN with surface and boundary layer properties is the absence of long‐term, continuous, and reliable ground‐based observations of sky condition over the remote continent. The methodology adopted in this study entails classifying the scene or sky condition at Dome C as clear, cloudy, or BLSN, with the help of CALIPSO satellite observations. This is followed by assembling and analyzing in situ surface and atmospheric observations at Dome C, that are nearly collocated and contemporaneous with respect to the CALIPSO overpass.

### 2.1 Scene Classification Using CALIPSO Measurements

CALIPSO uses lidar pulses for obtaining properties of the atmosphere, especially the vertical distribution of aerosols and clouds along a 100 m wide swath, while orbiting the earth (Winker et al., 2009). CALIPSO is in a sun‐synchronous orbit, with the orbit track repeating every 16 days. The CALIPSO Lidar Level 2 cloud layer product (1 km horizontal resolution; Liu et al., 2019) and the CALIPSO Lidar Level 2 BLSN product (333 m horizontal resolution; Palm et al., 2011) are used to identify cloudy and BLSN pixels, or CALIPSO shots, along the orbit in the vicinity of Dome C, respectively. A 3 year study period is selected, starting from April 2009 to March 2012, yielding a sufficiently large sample size for seasonal investigations.

**Figure 1:**
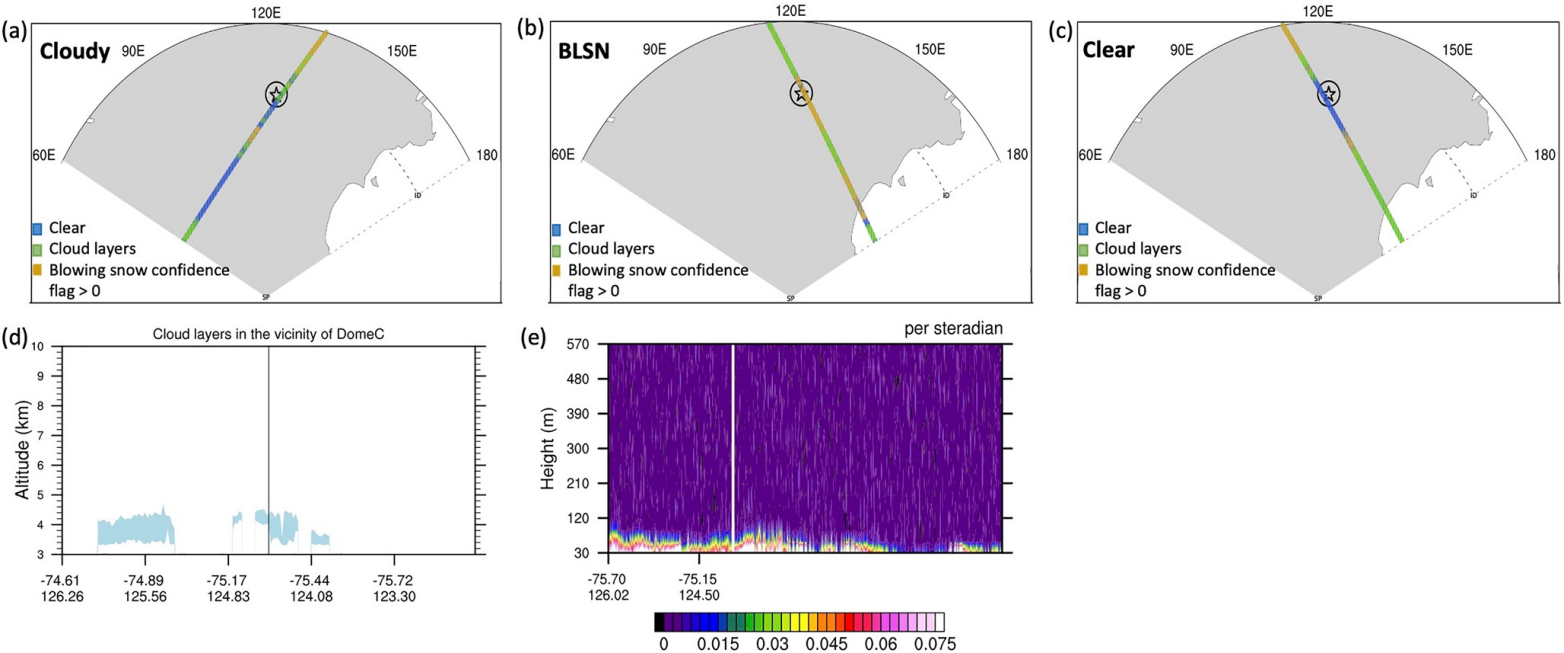
Scene identification at Dome C location based on Cloud‐Aerosol Lidar and Infrared Pathfinder Satellite Observations (CALIPSO) shots or pixel information for the CALIPSO track, which is the portion falling within the perimeter of 100 km radius (black circle) surrounding Dome C (black star), showing a typical (a) cloudy case on 31 July 2011, (b) blowing snow (BLSN) case on 02 July 2011, and (c) clear‐sky case on 16 July 2011. The scene identification takes into consideration the relative distribution of CALIPSO shots or pixels within the CALIPSO track, which are either clear (blue), having cloud layers (green), or having positive blowing snow (BLSN) confidence flag (gold). Bottom panel shows the height transect along CALIPSO track of (d) cloud layers (light blue shading) for the cloudy case, and (e) BLSN backscatter for the BLSN case. The pixel closest to Dome C is indicated by black vertical line and white vertical line in the respective transects.

For the period of study, the days on which the CALIPSO orbit passes within a 100 km radius surrounding Dome C are identified, and the portion of the orbit falling within this region (shown in Figure 1) is defined as “CALIPSO track” or simply “track.” For each month, there is an average of 19 such days when the above criterion is met, and the time of passage when CALIPSO pass is closest to Dome C varies between 15:07 and 15:37 UTC and/or between 07:25 and 08:01 UTC. Typically, there is one CALIPSO track per day, however, approximately every 16th day, there are two CALIPSO tracks that falls within 100 km from Dome C. The first occurs between 07:25 and 08:01 UTC and the latter between 15:07 and 15:37 UTC. The total number of individual CALIPSO tracks per month ranges from 18 to 22.

The classification method identifies cloudy cases as those with a cloud layer occurring at the CALIPSO pixel closest to Dome C and a requirement of negative BLSN confidence flag for all pixels along the CALIPSO track as seen in Figures 1a and 1d. (Negative values of BLSN confidence flag indicate no probability of BLSN occurrence. Positive values of BLSN confidence flag, ranging from 1 to 7, represent the retrieval quality for each pixel, considering wind speed, backscatter ratio, depolarization, surface elevation and other factors, with increasing values indicating more confidence of BLSN occurrence). Cases are classified as clear when there is negative BLSN confidence flag for all pixels along the CALIPSO track, and no cloud layer at the pixel closest to Dome C (as seen in Figure 1c). In addition, cases with 50% or more cloudy pixels along the CALIPSO track are excluded from the clear samples. In order to select BLSN cases, first and foremost, tracks with more than 10 pixels with positive BLSN confidence flag and at least one pixel with confidence flag greater than or equal to 2, are identified. Furthermore, the criteria for classifying as BLSN requires no cloud layer present at the pixel closest to Dome C as well as for 50% or more pixels along the CALIPSO track (the only exception is cases with low clouds i.e., cloud layer top height <1,000 m above surface as these are potentially BLSN cases that are misinterpreted as cloud by the cloud layer product algorithm). Figures 1b and 1e show a typical BLSN case. Cases with low confidence of BLSN, that is, maximum BLSN confidence flag for all pixels along the CALIPSO track <2, are disregarded. Cases satisfying the criteria for BLSN *and* cloud are also disregarded in an attempt to separate cloud and BLSN impacts. In addition to the above criteria for selecting BLSN cases, the backscatter profile associated with each BLSN event is manually inspected to check for false positives. False positives may occur due to various factors such as heterogeneity in terrain height or surface roughness, however, at Dome C, they are typically caused by solar background noise. Indeed, a majority of the false positives occur during November‐December‐January months when insolation is at peak, with 74% cases recorded as false positives during summer, 45% during spring, and only around 20% cases during fall and winter seasons, respectively. The false positive cases are identified by a thorough visual inspection of the data and are duly disregarded from our analysis.

### 2.2 Surface Observations at Dome C

The Baseline Surface Radiation Network (BSRN) station at Dome C (Driemel et al., 2018; Lupi et al., 2021) collects measurements of global, diffuse, direct, shortwave, and downwelling LW radiation every minute, in addition to air temperature, relative humidity, and atmospheric pressure at instrument height (i.e., 2 m). In this study, contemporaneous measurements of the mean downwelling LW radiation, air temperature, and pressure are noted at the hour and minute of satellite pass over Dome C that is, corresponding to CALIPSO track pixel closest to Dome C. In case of missing BSRN data at that particular time, validity of measurements falling within an hour window are checked and used when available. Valid surface measurements are typically available for more than 90% of the CALIPSO tracks and their corresponding scene classification during each season. Table 1 shows the total number of surface observations (temperature, pressure, and downwelling LW radiation) along with the mean and standard deviation of each quantity for different seasons and sky conditions. The results shown in Table 1 will be discussed in Section 3.

**Table 1:** Seasonal Averages and Standard Deviation of 2 m Air Temperature (T), Pressure (P) and Downward Longwave Radiation (LW) at Dome C, for Different Sky Conditions

	Summer			Fall			Winter			Spring		
	BLSN	Clear	Cloud	BLSN	Clear	Cloud	BLSN	Clear	Cloud	BLSN	Clear	Cloud
Mean T	**−41.43**	−34.5942	−33.0742	−64.2688	−63.2966	**−56.0808**	−66.637	−67.9623	**−57.6**	−55.9267	−54.281	−52.7
Stddev T	7.4241	7.8434	9.3498	6.0002	7.2188	10.2383	6.7667	7.8324	8.013	11.0893	10.8083	8.2969
#T	10	69	31	32	59	26	27	53	37	30	58	25
Mean P	**643.2**	650.676	**648**	641.3	640.034	**643.636**	**641.8148**	635.943	**642.27**	639.3	641.138	642
Stddev T	7.1926	7.2037	5.7038	6.2981	7.1389	7.6192	10.0194	11.5732	10.5215	8.7144	9.1909	10.0747
#P	10	68	31	30	59	22	27	53	37	30	58	25
Mean LW	94.5	102.775	**141.161**	71.6061	74.7705	**111.074**	65	64.4167	**103.867**	73	75.1034	**101.923**
StddevLW	6.9162	13.653	31.1406	9.5031	12.6628	31.5703	10	11.7275	33.9402	17.9828	15.0248	26.3058
#LW	10	71	31	33	61	27	28	60	45	30	58	26

*Note*. The number of observations used for calculation of mean, standard deviation, and significance levels are indicated for each variable. Statistically significant differences (95% confidence level or higher) compared to clear conditions are highlighted in bold.

### 2.3 Atmospheric Profiles at Dome C

Apart from surface data, daily upper air soundings are available at Dome C. Radiosonde launches take place at 12:00 UTC every day. The CALIPSO pass over Dome C occurs within a 3–4.5 hr window of the radiosonde launch. For days with single CALIPSO track, the daily radiosonde is classified as cloudy, BLSN, or clear based on the CALIPSO scene classification. For days with double CALIPSO tracks, the scene classification for the track closest to the time of launch is applied. The upper‐air data are checked for quality, and soundings with erroneous altitude readings and/or multiple readings at same altitude are ignored. The sounding on 29 May 2011, showed the presence of a shallow superadiabatic layer, which appeared as an outlier in our analysis, and is ignored. Similarly, the sounding on 04 April 2009 is disregarded due to erroneously high wind speed reading at the surface compared to upper levels (>500% magnitude). Overall, more than 95% of the soundings passed the quality check for use in this study. Note that due to missing radiosonde observations on random given days, the collocation with CALIPSO tracks yields a smaller sample size compared to that of collocated surface observations from BSRN.

In Section 3.1, all available radiosonde observations collected during the entire period of our study, are used to investigate the seasonal climatology of atmospheric conditions at Dome C. This is followed by using observations collocated with CALIPSO tracks for a detailed investigation of the relationship between clouds and BLSN with surface (Section 3.2) and boundary layer (Section 3.3) properties. Discussion and concluding remarks are made in Section 4.

## 3 Results and Discussion

### 3.1 Climatology of Dome C Atmospheric Profiles

**Figure 2:**
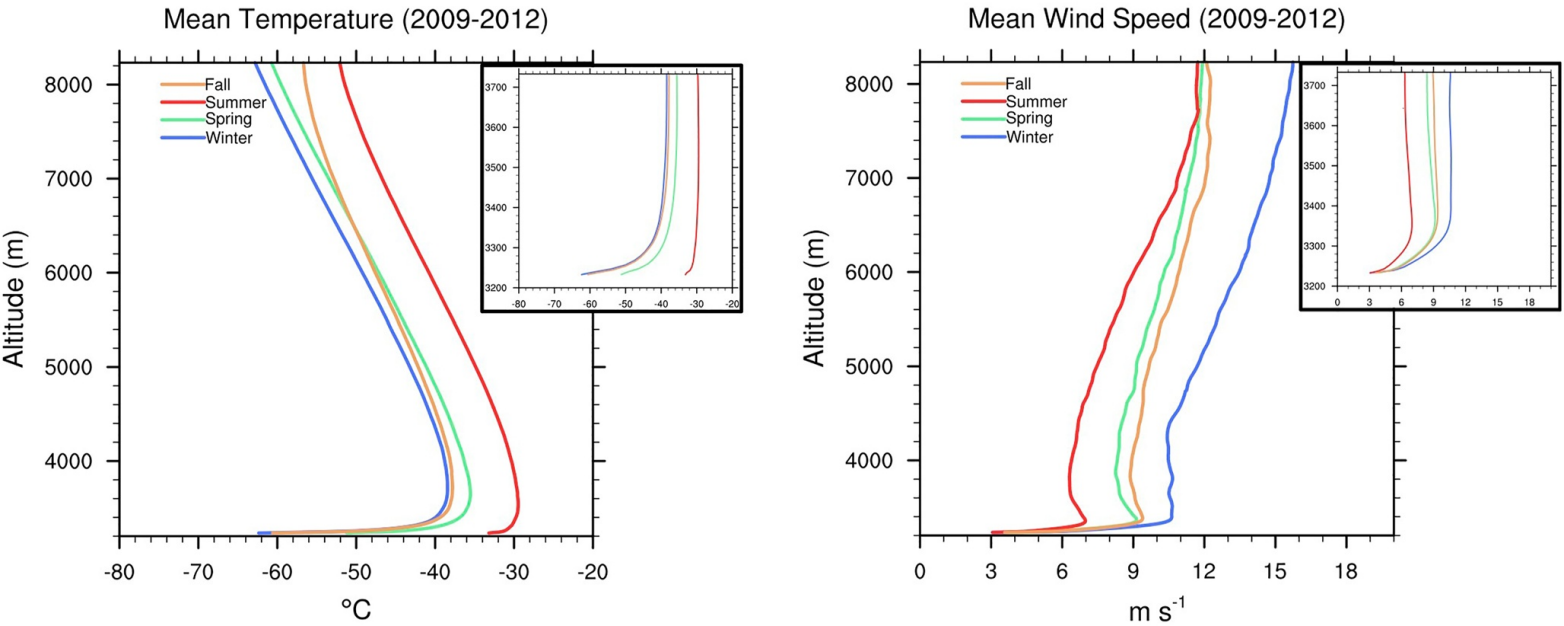
Seasonally averaged profiles of (left) temperature and (right) wind speed (inset zoomed in to lowest 500 m) for all 1200 UTC radiosonde observations collected at Dome C from April 2009 to March 2012.

Figure 2 shows the mean temperature and wind speed profiles for each season averaged for the period of our study, which includes all months and all days of valid radiosonde observations from April 2009 to March 2012. Fairly similar temperature profiles are observed during the cold season, especially fall and winter, and in contrast, the troposphere is significantly warmer during summer. Figure 2 confirms the occurrence of coreless Antarctic winter which has been noted in the past (Genthon et al., 2013; Town et al., 2007; Turner et al., 2009; Wendler & Kodama, 1993). When polar day concludes, the surface temperature quickly drops and is maintained from April through September, without a prominent “core” or monthly minimum that would normally occur around July‐August over southern hemisphere continental regions. Part of the reason for this is that snow over the Antarctic ice sheet has low thermal conductivity and heat capacity. The coreless winter does not occur over the Arctic, as unlike continental snow, the sea ice grows and thickens with the progress of the cold season. Over Antarctica, the coldest surface air temperatures and the most stable boundary layer conditions are consistently observed for fall and winter seasons (Figure 2).

**Figure 3:**
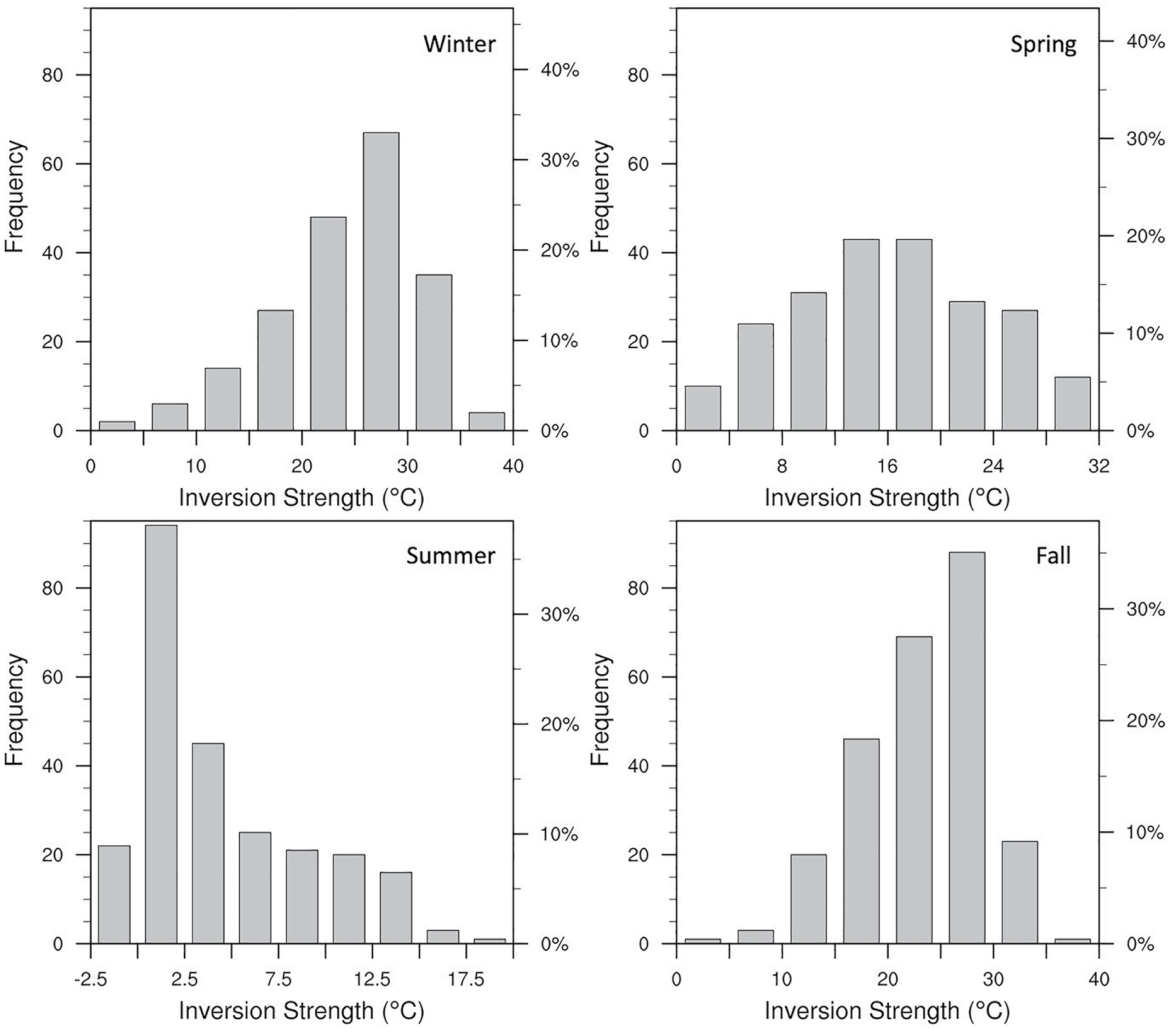
The frequency distribution of inversion strength for each season based on all 1200 UTC radiosonde observations collected at Dome C from April 2009 to March 2012.

Figure 3 shows the histogram distribution of the inversion strength calculated using the methodology based on Zhang et al. (2011). Assuming presence of a near‐surface inversion, the inversion layer top is computed using a bottom‐up search for the base of a layer, of at least 100 m thickness, with continuous negative temperature gradient. Barring summer season, an inversion is always found to be present. The inversion strength, computed as the difference in temperature between the inversion layer top and the surface, is always positive for fall, winter, and spring seasons. During summer, the inversion strength can be negative when no SBI is present. The median inversion strength peaks during fall and winter, as indicated by Figure 3.

The distribution of winter season inversion strength (upper left panel of Figure 3) agrees well with Petenko et al. (2019), who observed similar results during 2012 Dome C winter, with an average value of 23°C (24°C in our study), and maximum value between 35 and 40°C (38°C in our study). Petenko et al. (2019) attributed the negative skewness in the inversion strength distribution to moist airmass intrusions associated with synoptic episodes which occurred every 8–12 days (∼10% occurrence frequency) at Dome C in the winter of 2012, and were accompanied by strong low‐level wind speeds (up to 12 m s^−1^ at 3.6 m level), significantly higher surface air temperatures (up to −40°C), and cloudiness. Past studies have additionally found that these events are associated with higher surface pressure (Argentini et al., 2001; Petenko et al., 2009), and the positive anomalies of surface air temperature and pressure have both been attributed to the subsidence of moist elevated air advected from lower latitudes (Genthon et al., 2013). The negative skewness in the inversion strength distribution in the top left and bottom right panels of Figure 3, suggests that synoptic conditions during fall and winter seasons are likely similar to that described by previous Dome C studies (Argentini et al., 2001; Petenko et al., 2009, 2019), and will be investigated in Sections 3.2 and 3.3. On the other hand, the spring season inversion strength has no skewness in its distribution as well as a weaker mean value (∼16°C; top right panel of Figure 3). Note that the inversion depth distribution for spring season is similar to that of fall and winter seasons, with an average depth of ∼400 m (not shown). For summer, the distribution for inversion strength (and depth) are positively skewed, indicating that strong and deep inversions are a rare occurrence at Dome C in this season.

The wind speed profiles (right panel of Figure 2) suggest that low‐level maxima, occurring within the lowest kilometer, are present for each season. Summer has the calmest conditions with a mean low‐level wind speed maximum of ∼7 m s^−1^ observed around 125 m above the surface, followed by spring (9.1 m s^−1^ at 135 m) and fall (9.4 m s^−1^ at 154 m). The mean winter season profile has the strongest near‐surface winds with a maximum exceeding 10.6 m s^−1^ and occurring in a layer that is 160–560 m above the surface. During the 2012 winter season at Dome C, the maximum wind speed height was similarly observed to occur around 160 m (Petenko et al., 2019), whereas the 3.6 m level wind speeds were mostly characterized by calm (<4 m s^−1^) and moderate (4–6 m s^−1^) wind conditions, with 40% and 50% occurrence frequency, respectively.

### 3.2 Relation Between Sky Condition and Surface Properties

**Figure 4:**
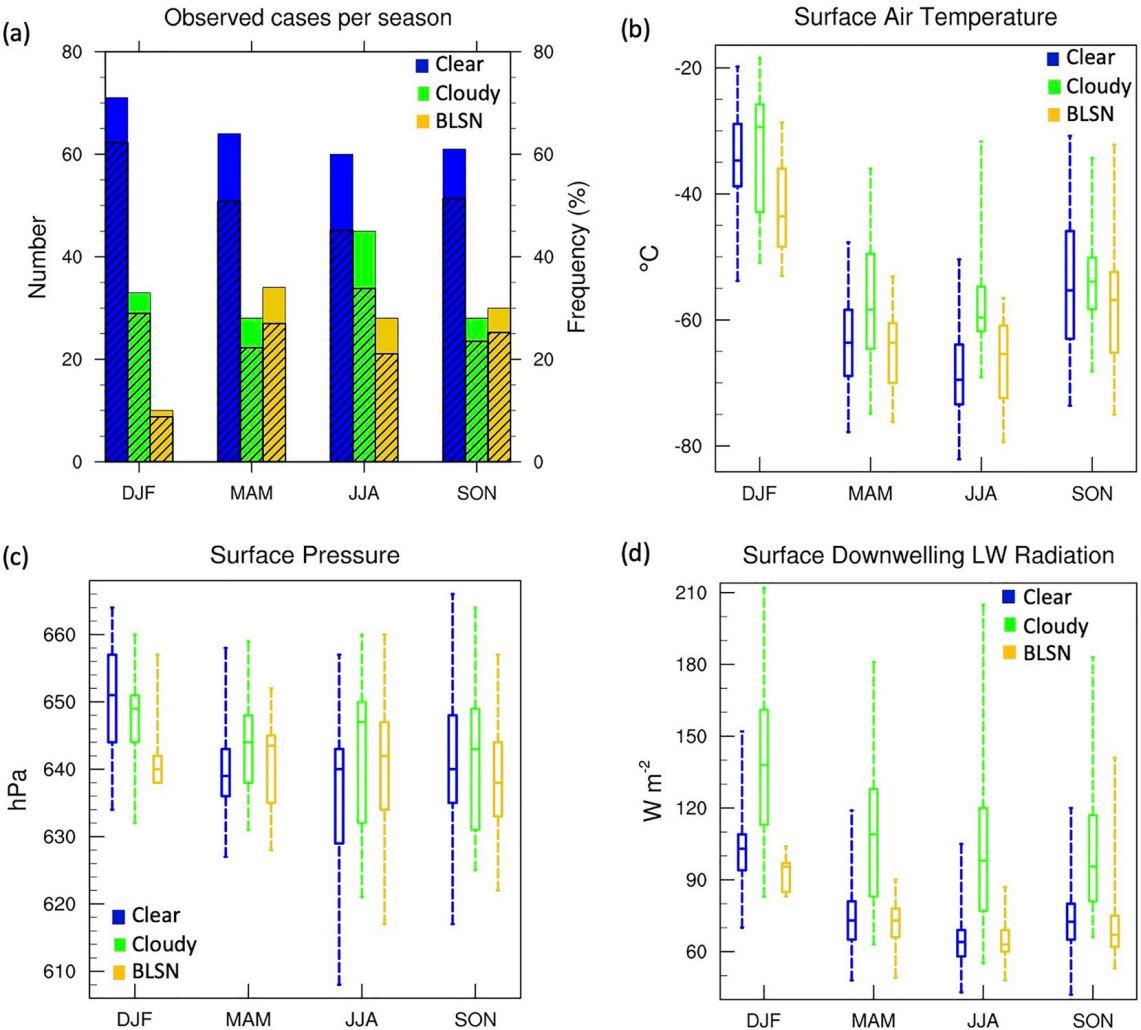
(a) Bar chart showing total number (shaded color; left axis) and frequency (shaded pattern; right axis) of clear, cloudy, and blowing snow (BLSN) cases based on Cloud‐Aerosol Lidar and Infrared Pathfinder Satellite Observations tracks passing over Dome C from April 2009 to March 2012. Box‐and‐whisker plot distribution of (b) surface air temperature, (c) surface pressure, and (d) surface downwelling longwave radiation from contemporaneous Baseline Surface Radiation Network measurements for clear, cloudy, and BLSN cases. The bounds of the box represent upper and lower quartiles, and the bounds of the whiskers represent the maximum and minimum values. The horizontal line in the middle of the box represents the median value.

Figure 4a shows the total number and frequency of cases based on CALIPSO scene type classification as explained in Section 2.1. Cloudy cases peak during winter (∼35% occurrence frequency), which is expected given the contribution from regular synoptic activity. BLSN cases occur with a frequency between 20% and 30% during fall, winter, and spring seasons, and less than 10% during summer (Figure 4a), in agreement with past observations (Palm, Kayetha, & Yang, 2018).

Figures 4b and 4c shows the seasonal distribution of surface air temperature and pressure from BSRN observations for each scene classification. The surface air temperature is higher during cloudy compared to clear‐sky conditions for all seasons, albeit, the difference is not always statistically significant (at the 95% confidence level) as seen in Table 1. For spring and summer seasons, cloudy cases are ∼1.5°C warmer than clear‐sky cases (Table 1), whereas, the warming is around 7° and 10°C, for fall and winter seasons, respectively. In fact, the surface air pressure also tends to be higher during cloudy cases in fall and winter seasons (Figure 4c; Table 1), therefore indicating that cloud formation largely occurs during the subsidence regime which comprises of moist, elevated airmasses that are advected from lower latitudes (Argentini et al., 2001; Petenko et al., 2009, 2019). Furthermore, during all seasons, the cloudy cases demonstrate significant increases in surface downwelling LW radiation (Figures 4d and Table 1), which may further contribute to the observed near surface warming, and will be explored in Sections 3.2.1 and 3.2.2.

For BLSN events compared to clear‐sky conditions, the surface is warmer during winter and cooler during the rest of the seasons, although the differences are not found to be statistically significant (Table 1). In the past, surface warming has been reported in association with BLSN events due to mechanical mixing in the presence of surface‐based inversions which transports warm air from top of the inversion toward the surface (Ganeshan & Yang, 2018; Palm, Yang, et al., 2018). This signature of surface warming, however, is not observed at Dome C, and the reason for the same is explored in Section 3.3.2. First, however, we will investigate the impact of clouds on the surface downwelling LW radiation and surface temperature in the following subsection.

#### 3.2.1 Impact of Clouds on Surface Downwelling LW Radiation and Temperature

**Figure 5:**
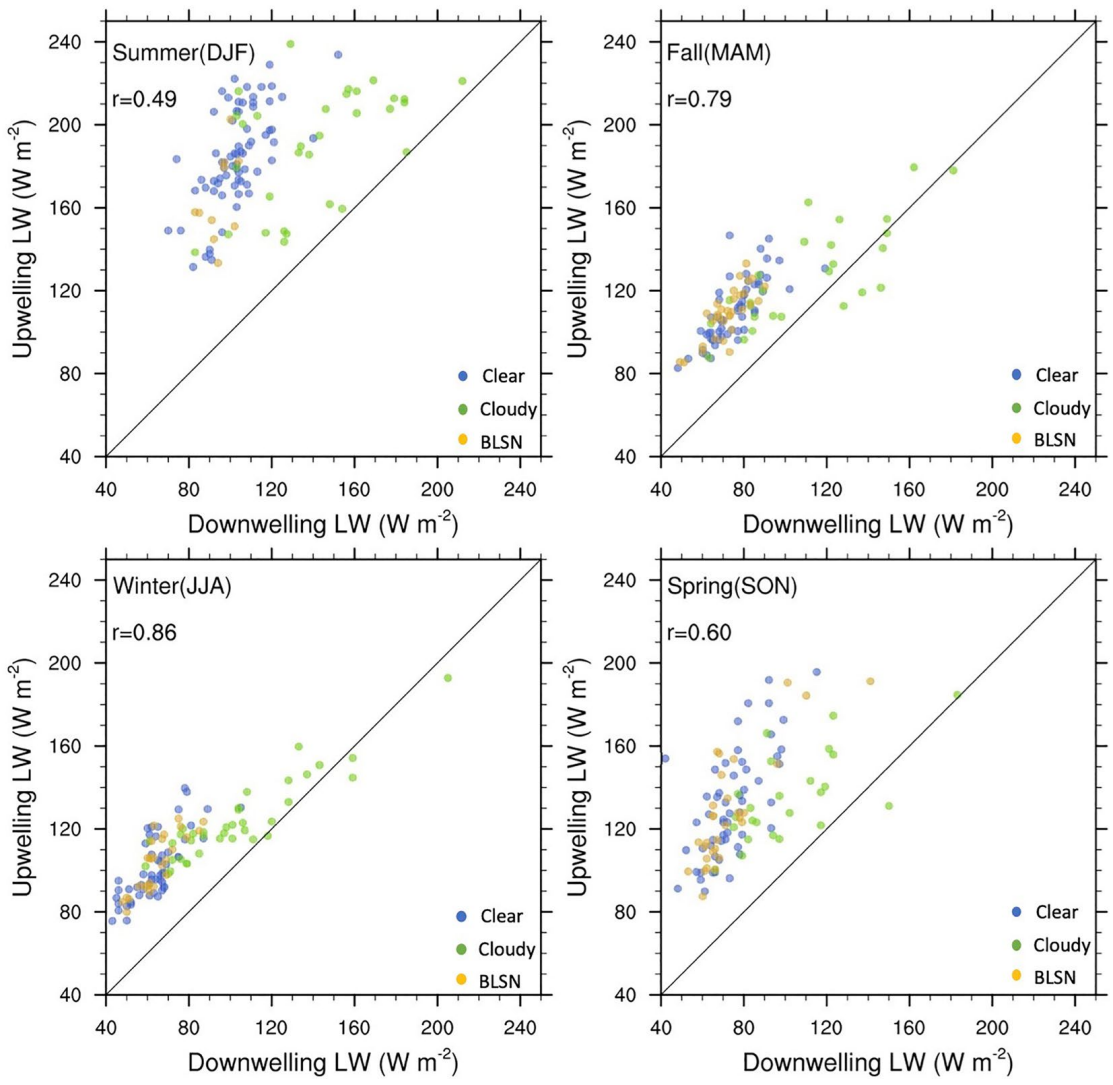
Scatter plot showing the relation between upwelling longwave (LW) radiation (estimated using Equation 1) and measured downwelling LW radiation at Dome C for different sky conditions and different seasons. Points falling on the black line, representing 1:1 correspondence, indicate radiative equilibrium.

As discussed in Section 1, Antarctic clouds can have a significant impact on the net surface radiation budget by trapping upwelling LW radiation and re‐emitting it back to the surface. Over the Antarctic icesheet, often there is an imbalance between the downwelling and the upwelling LW radiation, which results in surface cooling. The presence of cloud cover will lead to an increase in the downwelling LW radiation and warming at the surface. In order to investigate the radiative process at Dome C, the upwelling LW radiation is computed following the Stefan‐Boltzmann law:

(1) $$↑LW=\varepsilon_{s}\sigma T_{a}^{4}$$

where $\varepsilon_{s}≃1.0$ is the snow surface emissivity, T_a_ is the observed surface air temperature (measured at 2 m), and *σ* = 5.6696 × 10^−8^ W m^−2^ K^−4^ is the Stefan‐Boltzmann constant. When the upwelling and downwelling LW radiation are more or less equal, the surface is in radiative equilibrium. Figure 5 shows that during clear‐sky and BLSN conditions, the estimated upwelling LW radiation (Equation 1) is almost always larger than the measured downwelling LW radiation, indicating that the surface is not in radiative equilibrium, instead radiative cooling is dominant. Cloudy cases, for all seasons, are closer to radiative equilibrium (i.e., closer to 1:1 line in Figure 5) than clear‐sky or BLSN cases, suggesting that the enhanced downwelling LW radiation from clouds is likely countering the strong radiative cooling at the surface, thereby nudging it toward radiative equilibrium.

We can look at this from another perspective, that is, by investigating the relationship between the surface air temperature and the atmospheric column brightness temperature (*T*_*b*_) estimated from the measured downwelling LW radiation using Equation 2.

(2) $$↓LW=\sigma T_{b}^{4}$$

If the observed surface air temperature is warmer than the brightness temperature, it means that radiative cooling is dominant, whereas if they are approximately equal, it implies radiative equilibrium between the surface and the atmosphere.

**Figure 6:**
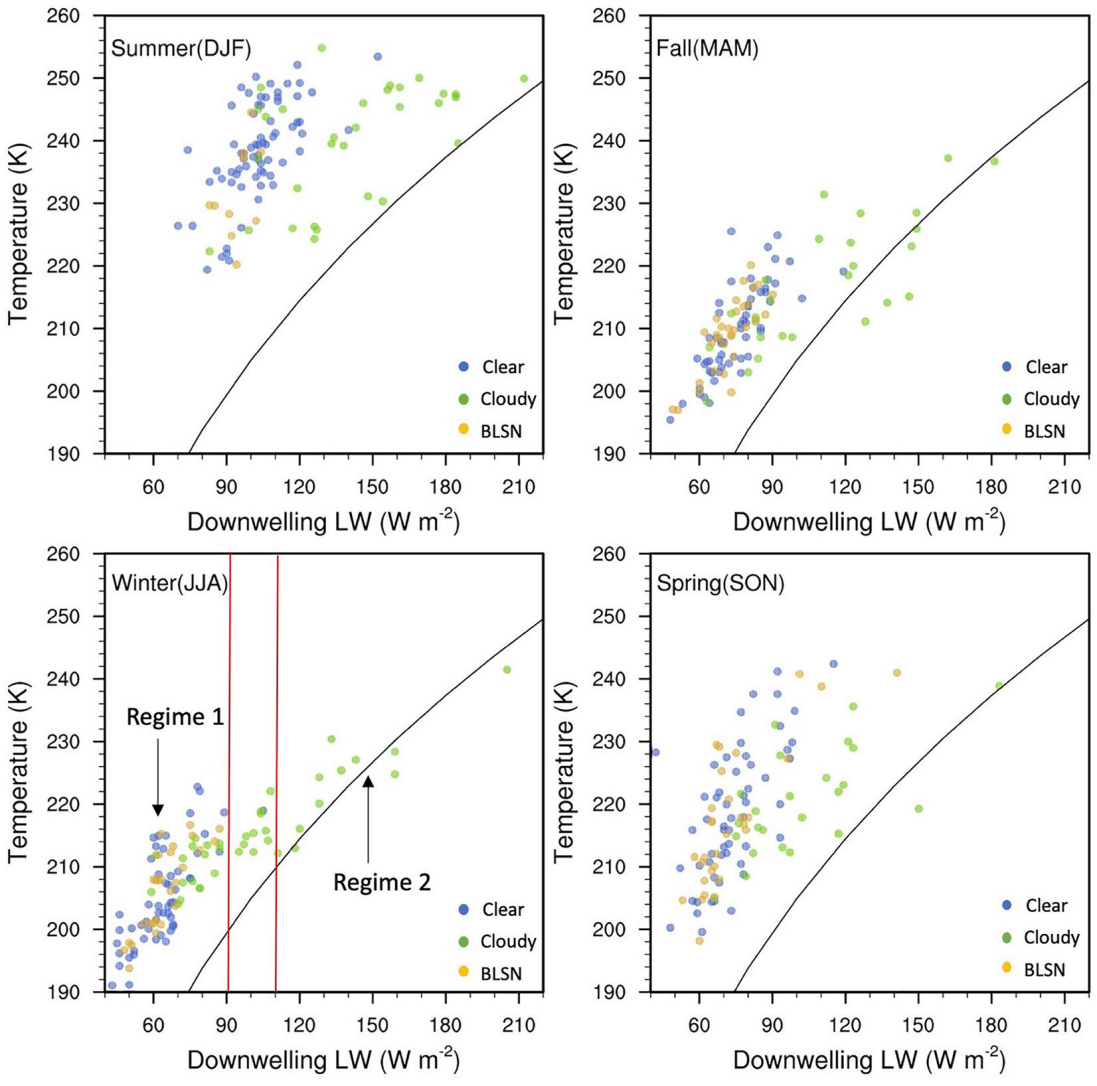
Scatter plot showing the relation between 2 m air temperature and downwelling longwave radiation at Dome C observed for different sky conditions and different seasons. Black curve is the corresponding brightness temperature of the atmospheric column (Equation 2), and points falling on it represent radiative equilibrium. The red lines are bounding intermediate values between Regime 1 and Regime 2 (see text for explanation).

Figure 6 shows the relationship between the surface air temperature and downwelling LW radiation (and corresponding atmospheric column brightness temperature computed using Equation 2) for each season and scene type. As observed by Hudson and Brandt (2005), there are two distinct regimes during winter (bottom left panel of Figure 6), both characterized by similar slope showing sensitivity of the surface air temperature to downward LW radiation. Cases in Regime 1 (values < 90 W m^−2^) occur to the upper left of the brightness temperature curve (i.e., observed surface air temperature values are higher than the estimated atmospheric column brightness temperatures), indicating dominance of radiative cooling at the surface. Cases in Regime 2 (values > 110 W m^−2^) more or less fall on the brightness temperature curve, suggesting radiative equilibrium at the surface. Hudson and Brandt (2005) speculated that this two regime behavior is related to the absence or presence of clouds. From Figure 6, it is confirmed that during winter, as well as other seasons to a large extent, radiative equilibrium at the surface is possible only during cloudy cases. On the other hand, whenever clear or BLSN cases are observed, radiative cooling is predominant. However, some cloudy cases also appear in Regime 1 (bottom left panel of Figure 6). For the middle range of values, between 90 and 110 W m^−2^ (between red lines in bottom left panel of Figure 6), the slope is flatter, which means that the surface air temperature is not as sensitive to the downwelling LW radiation. Hudson and Brandt (2005) showed that this behavior was associated with cases where the background conditions were rapidly changing, and a temperature‐radiation equilibrium was not yet achieved. It is worth noting that this intermediate phase of in‐equilibrium is also associated with clouds, suggesting that temperature changes at Dome C are likely caused by synoptic weather events that are typically accompanied by clouds (Argentini et al., 2001; Petenko et al., 2019). During summer, a more significant scatter suggests that there is less sensitivity of the surface air temperature to the downwelling LW radiation.

#### 3.2.2 Relationship Between Cloud Properties and the Surface LW Radiation Imbalance

As seen in Section 3.2.1, the near‐surface atmosphere at Dome C typically loses heat to its environment (due to the inequilibrium between downwelling and upwelling LW radiation), and clouds tend to reduce this net LW radiation imbalance by increasing the downwelling LW radiation and nudging the atmosphere toward equilibrium or even a net warming state. It is worth investigating if the reduction in net LW radiation imbalance is related to cloud properties, such as cloud thickness. The 1 km CALIPSO Lidar Level 2 Cloud Layer product provides the cloud layer base and cloud top heights, using which the number of cloud layers and the cloud layer thickness for single layer clouds, is estimated.

**Figure 7:**
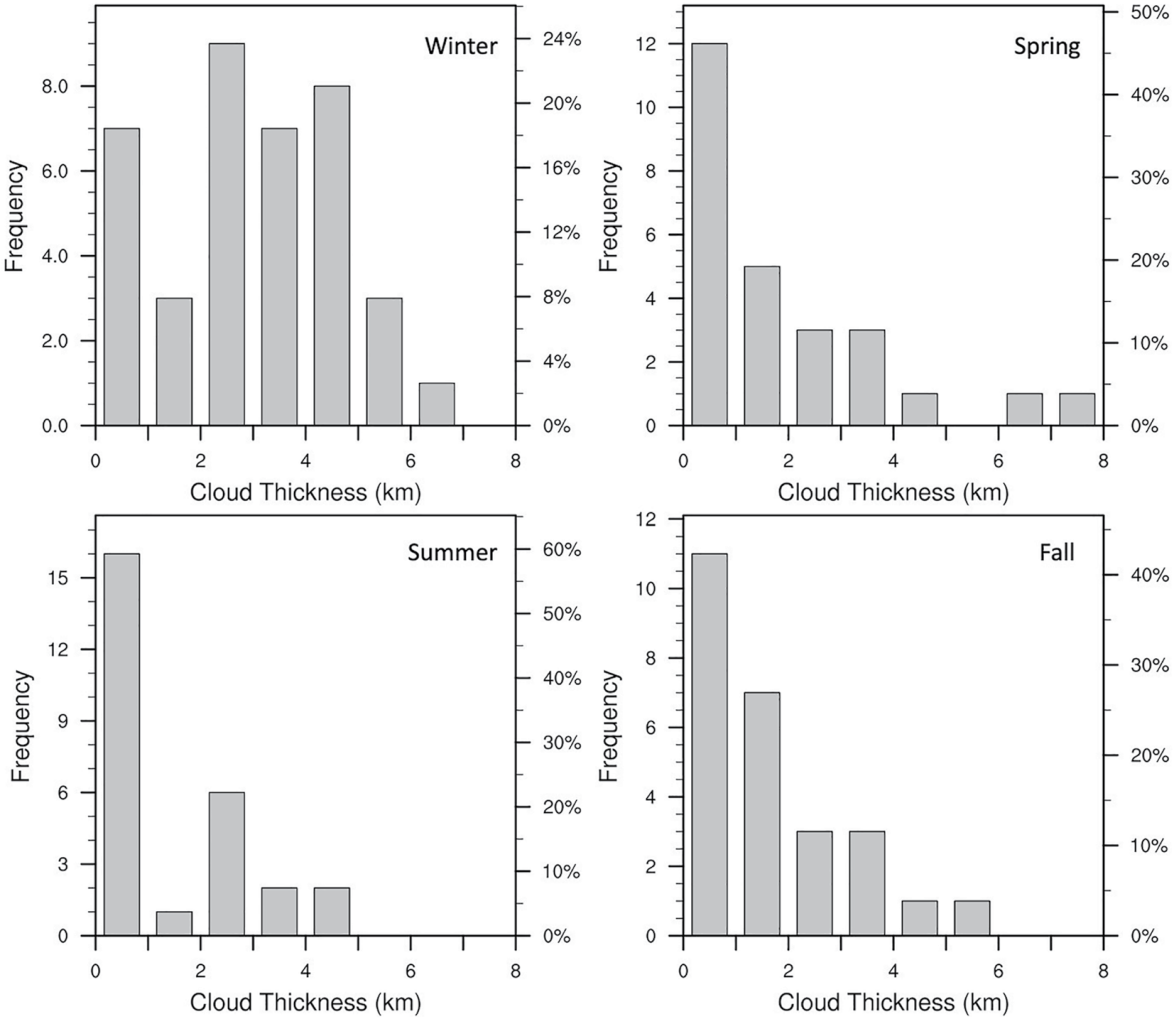
The frequency distribution of the geometric cloud thickness for single layer clouds at Dome C and for different seasons. Single layer clouds occur with a frequency of 85% in winter season, 93% in spring and fall seasons, and 82% in summer season, respectively.

Owing to its remote and elevated location, the atmosphere over Dome C is expected to be relatively dry. Cloud top heights are typically observed to occur within 4 km above the surface, except during winter season when clouds tend to extend into the mid‐to‐upper troposphere. The cloud base heights do not have any significant seasonal variability. Around 13% of the cloudy cases recorded at Dome C are multi‐layer clouds, and there is no evident seasonality in their occurrence. There are, however, seasonal differences in single layer cloud properties, specifically, their geometric thickness. As seen in Figure 7, thick single layer clouds are mainly observed only during winter (possibly aided by stronger synoptic disturbances compared to fall and other seasons). In general, there appears to be little diversity in cloud properties at Dome C, except for seasonal differences in cloud thickness (and cloud top height) described above. On the other hand, studies in coastal Antarctic regions, such as over the Ross Ice Shelf, McMurdo, and West Antarctic Ice Sheet, have found that cloud properties are highly variable and strongly linked with regional and synoptic atmospheric variability (Jolly et al., 2018; Silber et al., 2019).

Here we investigate the changes of LW radiation imbalance as a function of cloud thickness. For doing so, we first compute the normalized LW radiation imbalance, which is the normalized difference between downwelling and upwelling LW radiation, calculated using Equation 3.

(3) $$NormalizedLWradiationimbalance=\frac{↓LW-↑LW}{↑LW}$$

The downwelling LW radiation is from BSRN measurements, whereas the upwelling LW radiation is estimated from Equation 1.

**Figure 8:**
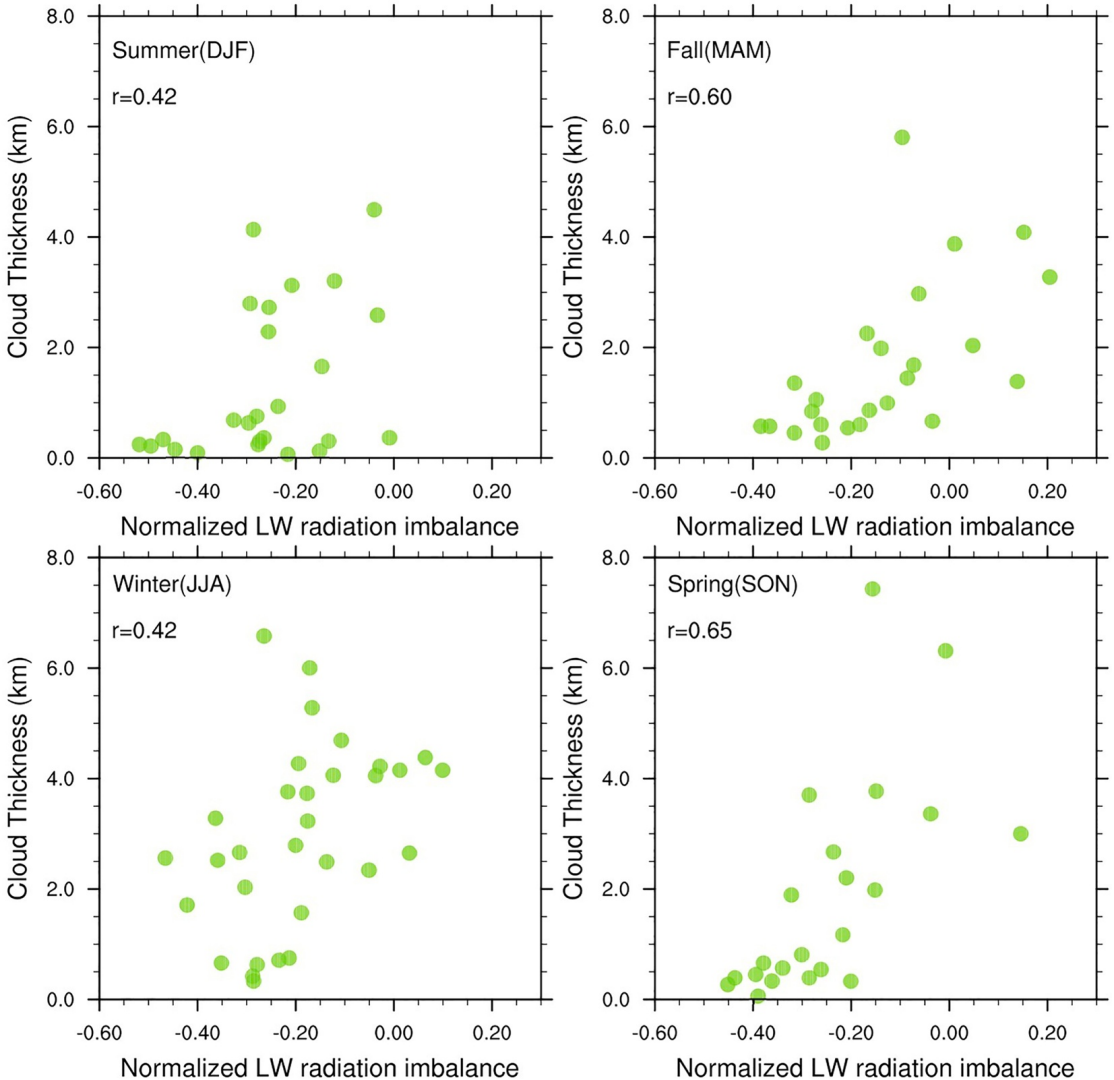
Scatter plot showing the relation between the geometric cloud thickness and normalized longwave (LW) radiation imbalance for single layer clouds at Dome C and for different seasons. The radiation imbalance (*X*‐axis) is the normalized difference between measured downwelling and estimated upwelling LW radiation (estimated using Equation 3).

Figure 8 shows that thicker the cloud, the more positive is the normalized LW radiation imbalance (i.e., more positive is the difference between measured downwelling and estimated upwelling LW radiation), suggesting that, at Dome C, thicker clouds contribute more efficiently toward reducing the LW radiation imbalance by nudging the near‐surface atmosphere toward radiation equilibrium and/or a net warming state. This is unequivocally observed for all seasons (Figure 8). As cloud optical thickness is strongly correlated with cloud geometric thickness, Figure 8 conclusively proves that surface warming observed during cloudy cases is indeed linked with the properties of the cloud.

### 3.3 Relation Between Sky Condition and Atmospheric Properties

**Figure 9:**
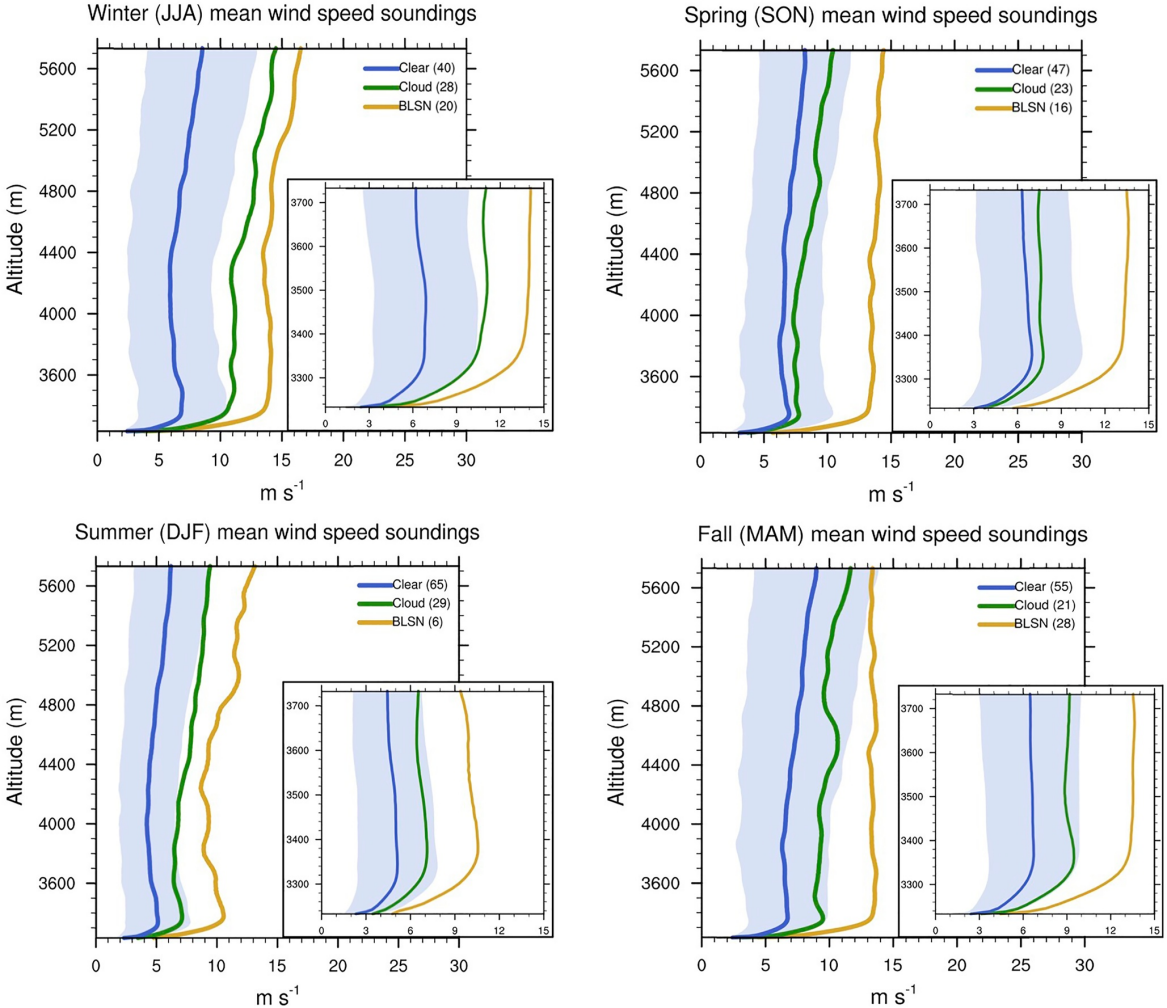
Seasonally averaged profiles of wind speed associated with clouds, blowing snow, and clear‐sky cases over Dome C (inset: zoomed in to lowest 500 m). Blue shading represents the uncertainty of wind speed measurements (i.e., the standard deviation associated with clear wind speed profiles).

**Figure 10:**
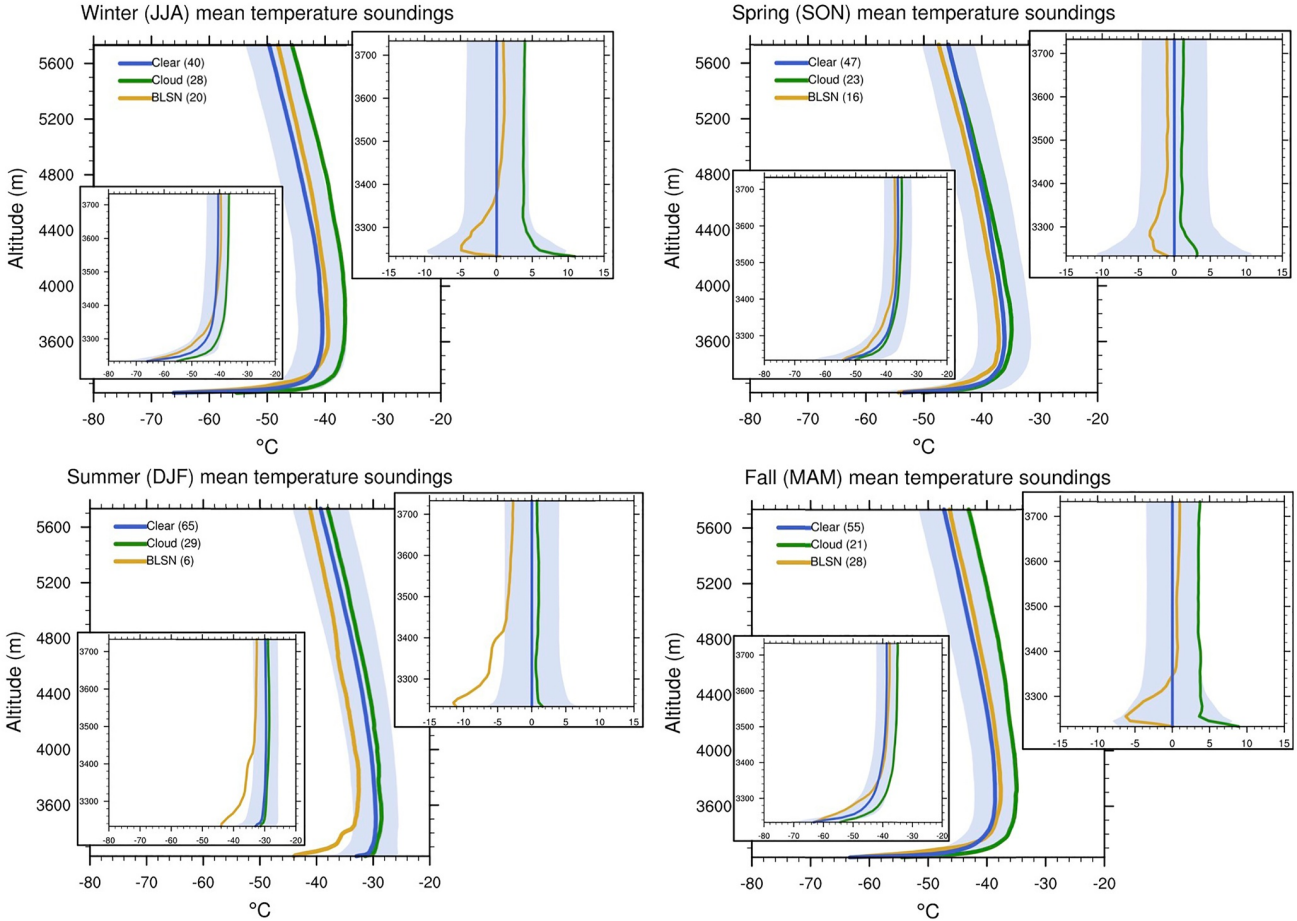
Seasonally averaged profiles of temperature associated with clouds, blowing snow, and clear‐sky cases over Dome C (left inset: zoomed in to lowest 500 m; right inset: difference with respect to clear‐sky in the lowest 500 m). Blue shading represents the uncertainty of temperature measurements (i.e., the standard deviation associated with clear temperature profiles).

**Figure 11:**
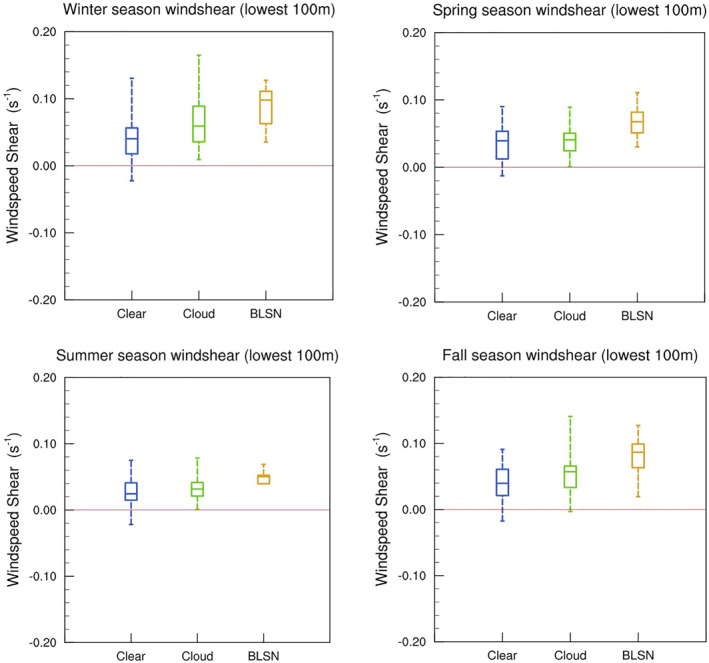
Box‐and‐whisker plot distribution of 100 m wind speed shear for clear (blue), cloud (green), and blowing snow (yellow) observed during different seasons at Dome C. The bounds of the box represent upper and lower quartiles, and the bounds of the whiskers represent maximum and minimum values. The horizontal line in the middle of the box represents the median value.

Figures 9 and 10 show the wind speed and temperature profiles for different seasons under different sky conditions. For all seasons, the maximum wind speeds occur during BLSN events. Cloudy cases are not as windy as BLSN, but when compared to clear‐sky cases, they have substantially higher tropospheric wind speeds for all seasons except spring. The distribution of low‐level wind speed shear (100 m level wind speed gradient with respect to the surface) confirms increased favorability for mechanical mixing for all (except spring) seasons during BLSN (cloud) occurrences (Figure 11). For BLSN cases, the signature of mechanical mixing is evident in the form of above‐surface cooling, however, in contrast to previous studies, there is no significant surface warming. For cloudy cases, the impact on temperature appears to be related to large‐scale meteorology (in addition to cloud LW forcing), because the accompanied warming typically extends to the entire troposphere, especially during winter and fall seasons (Figure 10). The impact of clouds and BLSN on the boundary layer thermal structure, and their seasonal differences, are explored in detail in the following subsections.

#### 3.3.1 Impact of Clouds on Atmospheric Profiles

**Figure 12:**
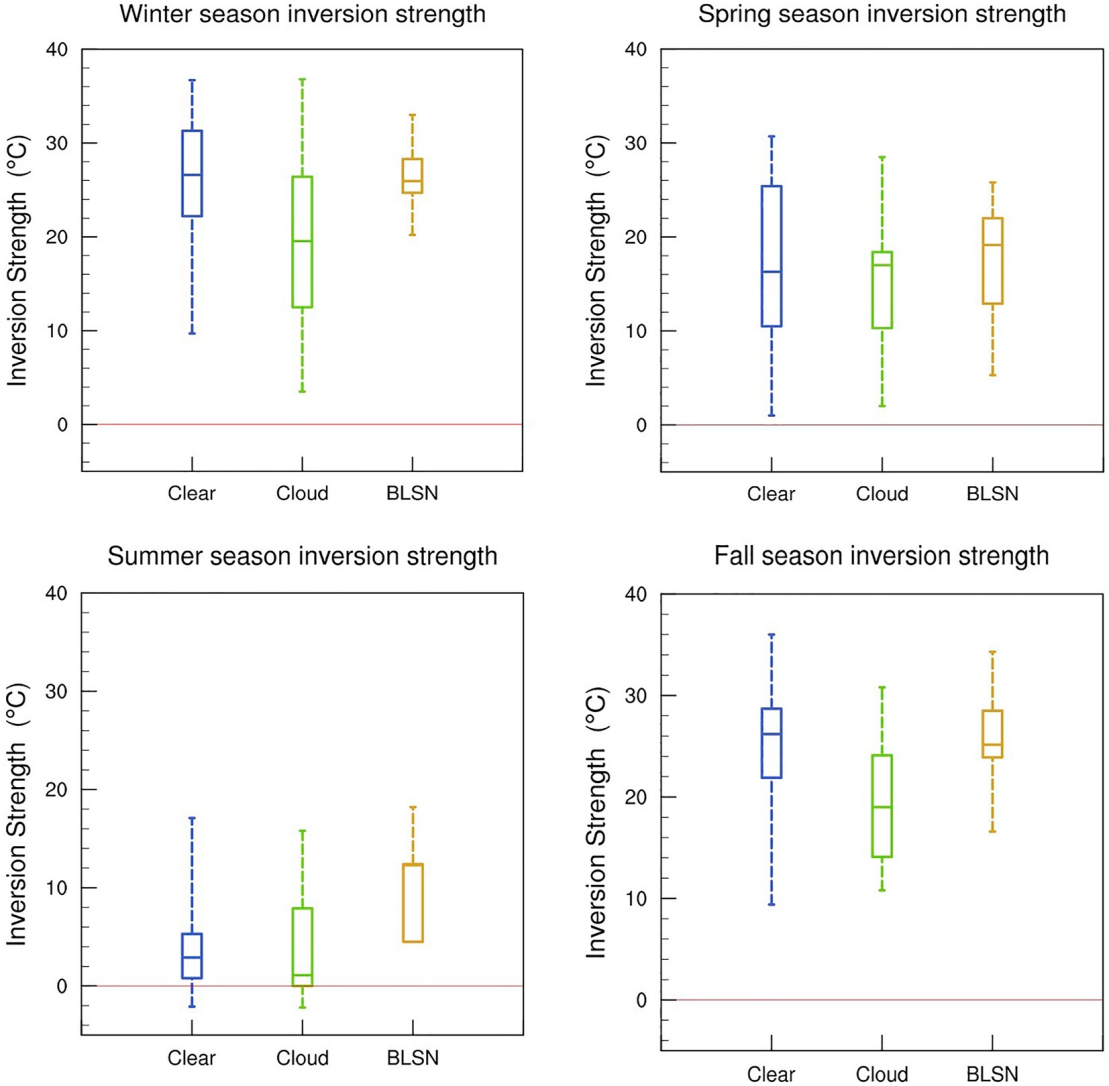
Same as Figure 11, but for inversion strength.

As seen in Table 1, cloudy conditions are associated with surface warming, which is most pronounced during fall and winter seasons. Figure 10 shows that clouds during both these seasons are also accompanied by a substantially warmer troposphere compared to clear‐sky conditions, indicating contribution from large‐scale subsidence (see discussion in Section 3.2), and moreover, culminating in a maximum temperature increase at the surface in the order of 10°C. Figure 12 shows the distribution of inversion strength for different sky conditions and each season. For fall and winter seasons, it is evident that the mean inversion strength during cloudy cases is significantly weaker than values observed during clear‐sky, by approximately 5° and 7°C, respectively. This, in addition to the evidence of stronger winds (Figure 9) and higher surface pressure (Table 1), confirms that fall and winter clouds are part of synoptic events that contribute to the negative skewness of the distribution shown in Figure 3 (as discussed in Petenko et al., 2019). The increased wind shear associated with these events (as seen in Figure 11) may additionally promote downward transport of warmer air, and thus contribute to surface warming via mechanical mixing. However, the lack of similar surface warming despite high wind shear observed during BLSN events in fall and winter (Figures 10 and 11) suggests that the contribution from mechanical mixing might be limited at Dome C. The dramatic surface warming observed in association with fall and winter clouds, thus, appears to come from large‐scale subsidence and LW feedbacks discussed in Sections 3.2.1 and 3.2.2.

For summer and spring seasons, the temperature profiles for cloudy cases do not differ substantially from that of clear‐sky cases, except for a near‐surface warming of around ∼3°C in spring and ∼1.5°C in summer (Figure 10). From Sections 3.2.1 and 3.2.2, it is clear that increased downwelling LW radiation observed during cloudy cases can abate radiative cooling losses and contribute to surface warming during all seasons, including spring and summer. The inversion strength distribution is more or less similar during cloudy and clear‐sky conditions (Figure 12). During spring, moreover, there are no significant differences in wind speed profiles (Figure 9) nor low‐level wind shear (Figure 11), suggesting that surface warming is likely caused by local cloud LW radiative feedbacks, as opposed to meteorology or mechanical mixing. For summer, Figure 9 indicates higher wind conditions during cloudy cases, with a low‐level maximum exceeding 7 m s^−1^ compared to only 5 m s^−1^ for clear‐sky. The temperature profiles, however, are not that different (Figure 10). There is a slight warming at the surface (∼1.5°C) when clouds are present (Figure 10), which is also observed in contemporaneous surface observations (Table 1). The downwelling impact discussed in Sections 3.2.1 and 3.2.2, expectedly muted during summer due to the compensating effect of shortwave cloud radiative forcing, is likely responsible for this warming.

#### 3.3.2 Impact of Blowing Snow on Atmospheric Profiles

For BLSN events occurring over Dome C, there is no indication of maritime or synoptic influences as the mean surface air temperatures (Table 1) and the median inversion strength (Figure 12) are comparable with that of clear‐sky cases (for all except summer seasons). All seasons show a significant increase in mean wind speeds (Figure 9) and wind speed shear (Figure 11) during BLSN events compared to clear‐sky, which is expected to result in substantial mechanical turbulence.

Figure 10 shows the consequent impact on the thermal structure of the atmospheric boundary layer. Two out of the six summertime blowing cases observed in our study show a negative temperature gradient (eroded SBI) at the surface, likely aided by mechanical mixing. Climatologically, BLSN events are less frequent during summer. It is therefore hard to conclusively quantify the impact on the atmospheric boundary layer given the small sample size (bottom left panel of Figure 10).

For fall, winter, and spring seasons, the SBI associated with BLSN events appears to be weakened (Figure 10). An above‐surface cooling is observed in the mean BLSN temperature profile compared to clear‐sky profile, which peaks between 20 and 30 m in fall and winter seasons, and around 50 m during spring season (Figure 10). This cooling is likely caused by shallow turbulence and the upward transport of radiatively cooled air from the surface. The corresponding warming at the surface, due to warm air being mixed downwards from the top of the inversion is expectedly mitigated due to the persistence of strong surface radiative cooling during such events (Figures 5 and 6).

The mean characteristics of this layer which exhibits cooling relative to clear‐sky profile (top inset Figure 10), is compared with that of the SBTL described by Petenko et al. (2019). During synoptically undisturbed conditions over Dome C, Petenko et al. (2019) observed a shallow layer of turbulence varying in depth from a few to several tens of meters with an average depth of ∼23 m, and occupying the lowest 3%–15% of the roughly 380 m deep SBI layer during winter. In our study, lower temperatures relative to the mean clear‐sky profile is observed during BLSN (top inset Figure 10), in a layer that is similarly embedded within the SBI, and occupies the lowest 3%–15% of the SBI depth. Petenko et al. (2019) further revealed that the SBTL height at Dome C is markedly lower than the height of the wind speed maximum of ∼160 m. As discussed in Section 3.1, the wind speed maximum for fall, winter, and spring seasons occurs above 130 m, whereas the cooling (and likely turbulence) evidently occurs within the lowest tens of meters from the surface (Figure 10).

**Figure 13:**
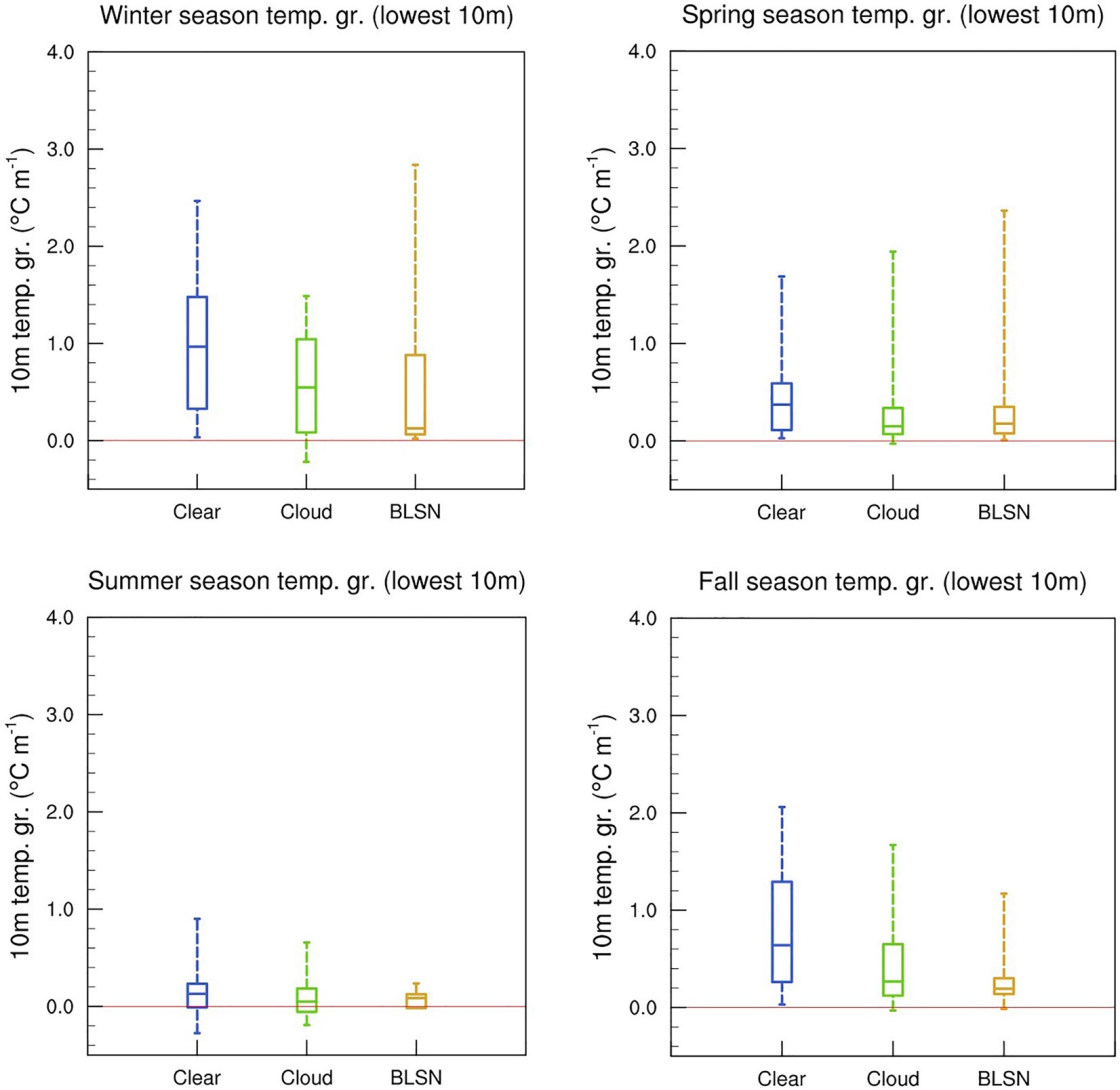
Same as Figure 11, but for 10 m temperature gradient.

Furthermore, from their measurements during wintertime synoptically undisturbed conditions at Dome C, Petenko et al. (2019) showed that the SBTL height was negatively correlated with the near‐surface temperature gradient (temperature difference between 10 m level and surface). In our study, the 10 m temperature gradient is strongest during clear‐sky conditions for all seasons (Figure 13). During winter and fall seasons, it is significantly weaker for BLSN and cloud cases (top left and bottom right panels of Figure 13), suggesting that atmospheric turbulence is strongly linked to wind‐driven shear (top left and bottom right panels of Figure 11). Indeed, the fall and winter 10 m temperature gradient are found to be negatively correlated with the low‐level wind speed shear (*r = −*0.35 and −0.41, respectively). For spring season (and summer), even though the low‐level wind shear is significantly higher during BLSN occurrences (top right and bottom left panels of Figure 11), there is no significant impact on the 10 m temperature gradient (top right and bottom left panels of Figure 13), likely because the BLSN related turbulence occurs within a deeper layer. This is evidenced by the higher peak in the relative cooling of the mean BLSN temperature profile during spring (top right inset Figure 10).

**Figure 14:**
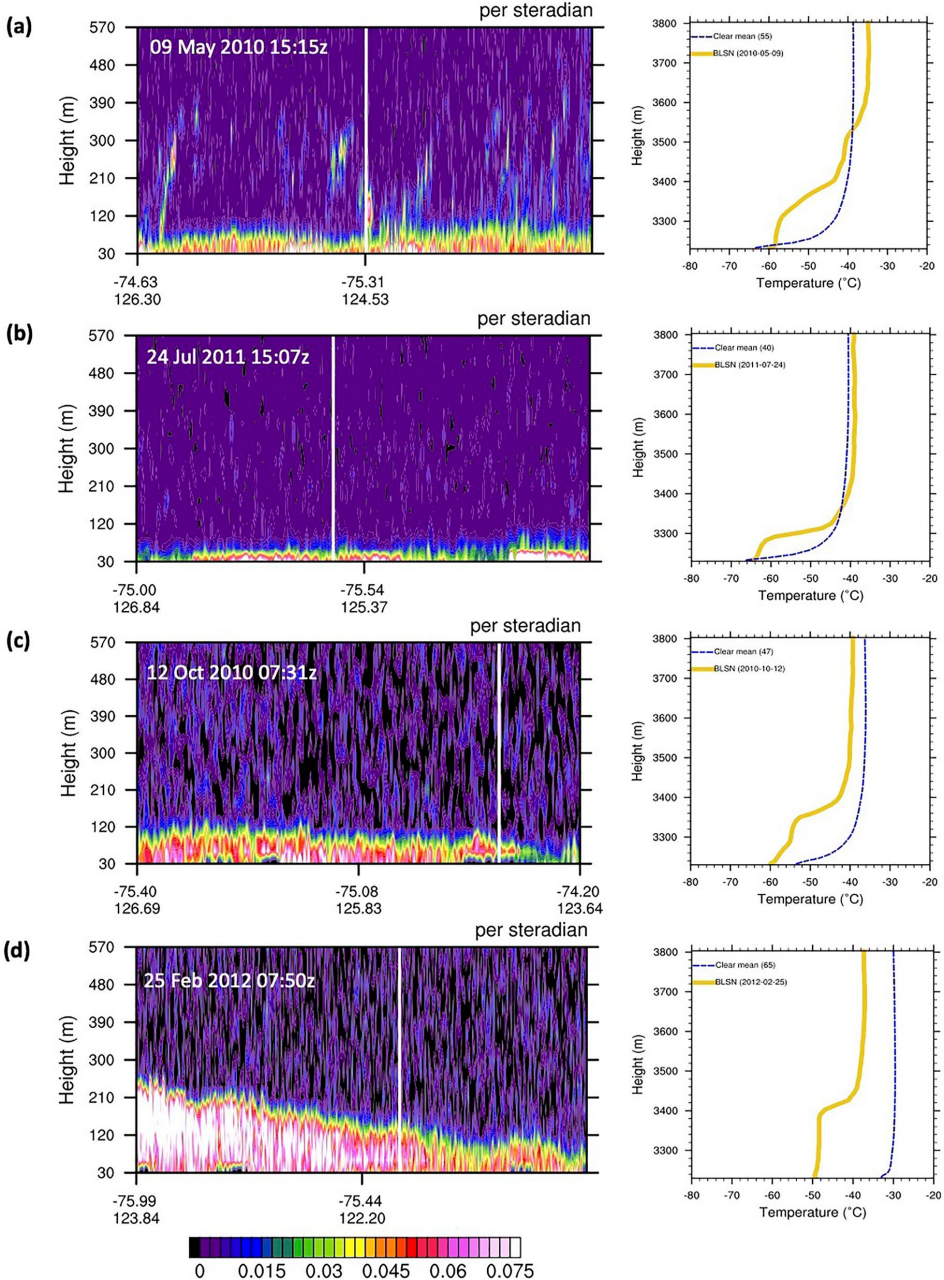
(left panel) Backscatter profiles of blowing snow pixels along the Cloud‐Aerosol Lidar and Infrared Pathfinder Satellite Observations track occurring in the vicinity of Dome C (location indicated by white line), and (right panel) same‐day 12z radiosonde temperature profile from surface to 570 m (yellow line) against the seasonal mean clear‐sky temperature profile (blue dotted line) on (a) 9 May 2010, (b) 24 July 2011, (c) 12 October 2010, and (d) 25 February 2012. Turbulent mixing is indicated by near‐isothermal temperature layer occurring above the surface (deeper for spring and summer cases compared to fall and winter).

Figure 14 shows backscatter profiles and radiosoundings associated with typical examples of BLSN occurrence at Dome C for each season. Deep turbulent mixing occurs during intense BLSN episodes, one of which occurred in summer, and led to the erosion of the SBI that is, formation of negative or isothermal temperature gradient at the surface (Figure 14d). This was consistently seen in the Palm, Yang, et al. (2018) study that utilized spring season dropsonde measurements to study the thermodynamic structure of well‐defined BLSN layers over the Antarctic interior. For the rest of the months, the inversion persists during BLSN events except for one case during late spring (similar to Figure 14d), and two cases during fall (not shown) where a very shallow (<10 m deep) negative temperature gradient layer occurs at the surface followed by a strong inversion above. Regardless, the signature of turbulence, as noted previously with respect to Figure 10, is evident in the form of above‐surface cooling and a consequent weakening of the positive temperature gradient in the lowest tens of meters. BLSN related turbulent mixing may indeed be identified by the near‐isothermal layer occurring above the surface and below the top of the inversion, embedded within the SBI (right panel of Figures 14a–14c), and termed as the turbulent layer. By the definition adopted in our study, the turbulent layer is a thermally perturbed layer connected to the surface and occurring below a layer with strong positive temperature gradient (an undisturbed inversion). The top of the turbulent layer is determined using a bottom‐up search for a strong, undisturbed inversion, that is, a layer of at least 10 m thickness with a positive temperature gradient exceeding 0.01°C m^−1^. The base of this layer is estimated as the height of the turbulent layer. For cases shown in Figures 14a–14d, the above definition yields turbulent layer heights of 78, 50, 110, and 170 m, respectively. Compared to spring and summer, the turbulent layer height appears restricted during fall and winter (typically <80 m) as seen in Figures 14a and 14b.

It is worth investigating if the BLSN layer depth, like the turbulent layer height, is restricted during fall and winter seasons. In the following section, the characteristics of the BLSN layer depth and its relationship with the turbulent layer are investigated.

#### 3.3.3 Blowing Snow Layer Depth and Its Relationship With Atmospheric Turbulence

**Figure 15:**
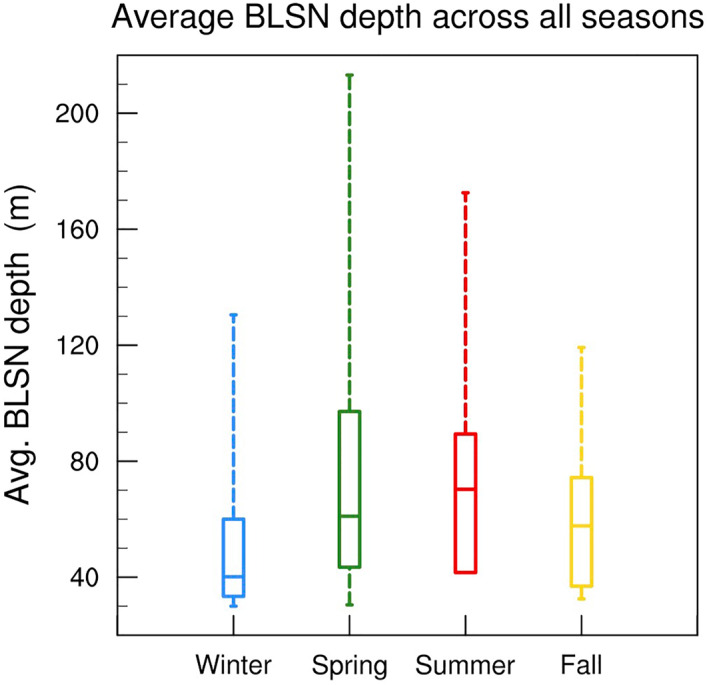
Box‐and‐whisker plot distribution of average blowing snow layer depth for different seasons at Dome C. The bounds of the box represent upper and lower quartiles, and the bounds of the whiskers represent maximum and minimum values. The horizontal line in the middle of the box represents the median value. Number of profiles for winter, spring, summer, and fall are 20, 16, 6, and 28, respectively.

Figure 15 compares the distribution of the average BLSN depth (i.e., the mean BLSN layer height averaged across all pixels with positive BLSN confidence flag across the CALIPSO track) for BLSN events in all seasons. The median of the average BLSN depth is lowest during winter (40 m) and highest during summer (70 m). The maximum of the average BLSN depth is substantially higher (213 m) during spring compared to winter and fall as indicated by the long tail in its distribution (Figure 15), indicating a seasonal dependence, similar to that of the turbulent layer described in Section 3.3.2.

In general, the background stability seems to be an important factor influencing the BLSN layer depth. When the inversion strength is greater than 24°C, majority of the BLSN events are restricted to less than 80 m average depth. Moreover, deep BLSN events (>100 m average depth) only occur when the inversion strength is less than 30°C. The mean inversion strength is significantly weaker during spring and summer, compared to fall and winter (Figure 3), which might explain the observed seasonal differences in the BLSN depth distribution (Figure 15).

**Figure 16:**
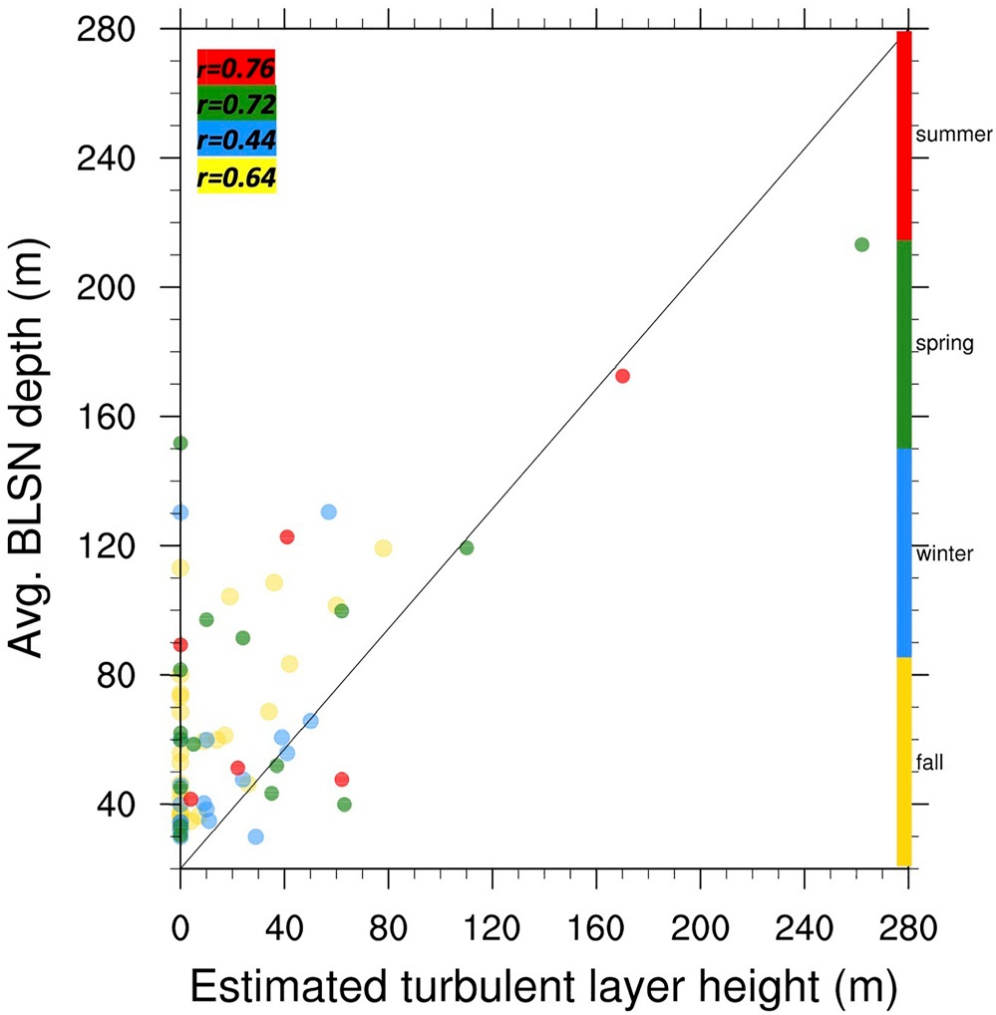
Scatter plot showing the relation between the estimated turbulent layer height (*X*‐axis) and the average blowing snow depth (*Y*‐axis) for summer (red), spring (green), winter (blue), and fall (yellow) seasons. The correlation coefficient “*r*” is provided for each season.

The relationship between the average BLSN depth and the height of the turbulent layer estimated using the method described in Section 3.3.2, is investigated with the help of Figure 16. Blowing Snow at Dome C is less frequent (Palm, Kayetha, & Yang, 2018) and when it occurs, it is likely to be less intense compared to its surrounding regions owing to the elevation and relatively flat terrain at Dome C. As a consequence, BLSN events occurring along the CALIPSO track may not always show a significant impact on the Dome C radiosonde temperature profile. For example, Figure 16 shows that for many of the BLSN events considered in our study, the estimated turbulent layer height at Dome C is zero regardless of the average BLSN depth (which is computed along the CALIPSO track), implying that the surface temperature gradient at Dome C can remain strongly positive (relatively undisturbed) when BLSN is occurring in the immediate vicinity. This does not mean that BLSN related turbulence is non‐existent, but simply that the BLSN is likely weaker in intensity at Dome C location (compared to its surrounding CALIPSO pixels) and therefore lacking a clear thermal signature. Note that strong BLSN events are typically accompanied by non‐zero turbulent layer height at Dome C and are always characterized by the presence of weaker SBIs and/or near‐isothermal layers, similar to that observed by Palm, Yang, et al. (2018) during the Concordiasi campaign. Figure 16 indeed shows an overall positive correlation between the observed BLSN layer depth and the estimated turbulent layer height for all seasons. For the deepest observed BLSN event (depth of 213 m), the estimated turbulent layer height exceeds 260 m. In fact, relatively weaker background stability (inversion strength <10°C) appears to favor deeper BLSN and atmospheric turbulence in this case. In other words, the background stability (inversion strength) may influence the vertical extent of atmospheric boundary layer turbulence as well as the maximum BLSN depth (Figure 16).

## 4 Summary and Conclusions

Clouds and BLSN are commonly occurring phenomena over Antarctica, and knowledge regarding their contribution to the surface mass balance and surface radiation budget are paramount for accurately modeling Antarctic climate. In the past, studies have focused on radiative feedbacks, however, the relationship of Antarctic clouds with the atmospheric boundary layer is less explored. Similarly, isothermal or well‐mixed boundary layer structure during Antarctic BLSN occurrences was previously recorded during austral spring season, however, the nature of the stable wintertime atmospheric boundary layer during BLSN events was unknown. Dome C, an elevated peak in East Antarctica, is useful for studying cloud and BLSN interactions with the surface and stable atmospheric boundary layer, because of the availability of long‐term satellite and in situ measurements. One drawback, however, is that BLSN occurs less frequently, and often times with seemingly weaker intensity, over Dome C compared to its surrounding areas. In order to capture sufficient and reliable cases for statistical analysis, 3 years of CALIPSO cloud and BLSN products (from April 2009 to March 2012) are used for the classification of the sky condition over Dome C as clear, BLSN, or cloudy. Co‐located and contemporaneous in situ measurements collected closest to the CALIPSO pass over Dome C are investigated for each classification type. The in situ data include surface downwelling LW radiation, surface air pressure, and surface air temperature observations from the BSRN, as well as upper air measurements of wind speed and temperature from daily radiosoundings.

For all seasons, there is a significant increase in the measured downwelling LW radiation during cloudy cases compared to clear‐sky and BLSN cases. This increase abates radiative cooling losses, contributing to surface warming. During spring, this surface warming amounts to around 3°C that appears to be caused primarily by cloud downwelling LW radiative feedback. The surface warming during fall and winter is even more dramatic (in the order of 10°C), however, in addition to cloud downwelling LW radiative feedback, it appears to be caused by large‐scale subsidence associated with synoptic events.

During BLSN events, the low‐level wind speed and wind speed shear are a maximum for all seasons. The inversion is often eroded during summer (i.e., a negative temperature gradient is observed at the surface), which may occur not only due to enhanced wind‐driven turbulence, but also due to insolation and related surface heating. For fall, winter, and spring seasons, the SBI typically persists but is weakened when BLSN occurs. For several BLSN cases, including the strongest events, a near‐isothermal layer is observed above the surface. The signature of atmospheric turbulence is evident through weakening of the SBI strength due to above‐surface cooling (often associated with the near‐isothermal layer). The height of the layer with weakened SBI is positively correlated with the BLSN layer depth for all seasons, and both layers are found to be shallower in winter and fall seasons owing to the stronger background stability.

This investigation has brought to light the nature of the relationship of Antarctic clouds and BLSN with the stable atmospheric boundary layer that tends to persist over Dome C. One caveat is that ground‐based observations of BLSN are not available at Dome C location hence it cannot be guaranteed that BLSN was indeed occurring at Dome C. In an effort to mitigate uncertainty in BLSN observed by CALIPSO, only cases with higher confidence of BLSN occurrence were analyzed. While this study laid emphasis on seasonal aspects, our approach can be expanded to include additional years of observations at Dome C, spanning more than a decade of CALIPSO and in situ measurements, that will allow investigation of interannual and other modes of variability. Importantly, this work lays the foundation for future studies using additional sensors, such as the Ice, Cloud, and land Elevation Satellite (ICESat‐2), which has the ability to point to ground targets and provides a BLSN product similar to CALIPSO (Palm et al., 2021). The ability of ICESat‐2 to provide exactly (geographically at least) coincident data will greatly aide studies of BLSN impact on the atmospheric boundary layer.

## Erratum

In the originally published version of this article, the ninth sentence of the fourth paragraph of section 3.2.1 contained a typographical error involving repetition of the word “presence.” The errors have been corrected, and this may be considered the authoritative version of record.

## Data Availability

The CALIPSO 1 km cloud layer product data used in this study are from NASA's Earth Observing System Data and Information System (EOSDIS) Distributed Active Archive Centers (DAAC) which can be accessed here https://earthdata.nasa.gov/eosdis/daacs. The CALIPSO Blowing Snow data are publicly available from the NASA Langley Atmospheric Science Data Center (ASDC) at https://earthdata.nasa.gov/eosdis/daacs/asdc. The BSRN data are from WRMC, and can be downloaded from http://www.bsrn.awi.de/. The radiosonde data are from IPEV/PNRA Project “Routine Meteorological Observation at Station Concordia” (http://www.climantartide.it).
